# Use of Fungal Mycelium as Biosupport in the Formation of Lichen-like Structure: Recovery of Algal Grown in Sugarcane Molasses for Lipid Accumulation and Balanced Fatty Acid Profile

**DOI:** 10.3390/membranes12030258

**Published:** 2022-02-24

**Authors:** Savienne Zorn, Ana Carvalho, Heitor Bento, Bruno Gambarato, Guilherme Pedro, Ana da Silva, Rhyan Gonçalves, Patrícia Da Rós, Messias Silva

**Affiliations:** 1 Engineering School of Lorena, University of São Paulo, Lorena 12602-810, SP, Brazil; anacarvalho@usp.br (A.C.); guilhermepedro@usp.br (G.P.); eng.ana.teixeira@usp.br (A.d.S.); patriciadaros@usp.br (P.D.R.); messias.silva@usp.br (M.S.); 2 Institute of Chemistry, Federal University of Alfenas, Alfenas 37130-001, MG, Brazil; rhyan.goncalves@sou.unifal-mg.edu.br; 3 Faculty of Pharmaceutical Sciences, São Paulo State University, Araraquara 14800-903, SP, Brazil; heitor.bento@unesp.br; 4 Department of Engineering and Technology, University Center of Volta Redonda—UniFOA, Volta Redonda 27240-560, RJ, Brazil; bruno.gambarato@foa.org.br; 5 Faculty of Engineering, Paulista State University Júlio de Mesquita Filho—UNESP, Guaratinguetá 12516-410, SP, Brazil

**Keywords:** lichen-like structure, *Scenedesmus obliquus*, *Mucor circinelloides*, mature mycelium, fatty acid composition

## Abstract

In this study, a lichen-like structure was obtained through the production of a unique biomass, formed by algae cells of *Scenedesmus obliquus* adhering to the mycelium of filamentous fungal *Mucor circinelloides.* This structure was composed in two steps; in the first one, microalgal cells and spores were incubated separately, and in the second one, after 72 h of growth, isolated, mature mycelium was harvested and added to cell culture. For spores’ incubation, a culture medium containing only 2 g·L^−1^ of glucose and minerals was used. This culture medium, with low sugar content, provided a fungal biomass to the anchorage of microalgae cells. WC medium was used without and with sugarcane molasses supplementation for microalgae cells’ incubation. The lichen-type structure that was formed resulted in 99.7% efficiency in the recovery of microalgae cells and in up to 80% efficiency in the recovery of algae biomass in the lichen biomass composition. In addition, the resulting consortium attained a satisfactory lipid accumulation value (38.2 wt%) with a balanced fatty acid composition of 52.7% saturated plus monounsaturated fatty acids and 47.4% polyunsaturated fatty acids. Since fungal species are easy to recover, unlike microalgae, the lichen-like structure produced indicates an efficient low-cost bioremediation and harvesting alternative; in addition, it provides an oleaginous biomass for various industrial applications.

## 1. Introduction

In recent decades, the development of renewable fuels and other bioproducts of high value and commercial interest has been intrinsically linked to technologies involving the processing of microalgae [1]. Microalgae synthesize biomass that is rich in valuable products such as lipids, proteins and carbohydrates; thus, their adaptability to diverse conditions, including environmental conditions, and their capacity to modulate cellular metabolism, result in high-value compounds that include carotenoids, chlorophylls, phycobiliproteins and polyunsaturated fatty acids [2]. Among the profitable derivatives of microalgae, some target metabolites stand out, such as astaxanthin, in which some algae species, including *Haematococcus pluvialis*, are a natural supplier [3]. In addition, being linked within a circular economy context, the employment of microalgae as a feedstock for clean energy generation has made tangible progress in recent years [4]. 

The use of microalgae can go far beyond their role as new providers of microbial lipids [5], which can turn into valuable methyl and ethyl esters for the biodiesel industry [6]; they can, in fact, function as a platform to capture CO_2_ and, indirectly, convert it into liquid fuel and other high-commercial-value bioproducts. However, the operational facilities for microalgae production still present some limitations, such as a very high demand of energy and the enforcement of requirements related to the use of toxic or recalcitrant chemicals in the harvesting steps [7]. Thus, the harvesting of tiny microbial cells from large cultures can account for 50% of the total biodiesel production cost [8].

Among the challenges to be overcome, water and nutrient requirements still represent a drawback due to the ecological implications of algae’s application in natural contexts. Microalgal cells are of small size and present specific gravity values similar to those presented by their culture medium, which are not characteristic, for example, of fungal species [9]. Owing to this peculiarity, microalgae, if harvested using traditional methods, are associated with costly operations [8]. It is notorious that microalgae are microorganisms that are able to interact mutualistically with other microorganisms, such as bacteria, yeasts, other microalgae as well as filamentous fungi [5,8]. Specifically in terms of the interaction between microalgae and filamentous fungi, it is possible to obtain the formation of consortia, as demonstrated by different researchers [10,11]; in such cases, interactions can favor the synthesis of compounds that are promising for industrial bioprocesses [11,12,13]. Based on this premise, it is possible to establish a biotechnology approach that involves different microbial species [14], blended with the use of the most diverse available raw materials [4].

Microalgae grow by converting energy into food through photosynthesis, but they can also develop mixotrophically or heterotrophically, as many species are robust and adapt well to different conditions and culture media [15,16]. On the other hand, some filamentous fungi can act as attractive bioflocculants due to their self-pelletizing nature, by which microalgal cells are attracted and trapped [5,8,11].

Among various biorefineries’ feedstocks, sugarcane molasses (SM) stand out as a valuable by-product. SM are broadly produced in Brazil, as well as in other countries such as India, Thailand, China and Pakistan, and are used as raw materials or as rich supplements in different segments inside and outside of biorefineries [17,18,19]. The composition of SM resembles that of sugarcane juice and, after sugar processing (sucrose crystallization), still presents a high concentration of sucrose (non-crystallized), glucose, fructose and also minerals, vitamins and trace elements [18]. Thus, being low-cost raw materials, SM could be applied as new inputs in biorefineries, contributing to the integration of processes. Oleaginous fungus and oleaginous microalgal biomass, also named Single Cell Oil (SCO), are a very interesting product, since microbial oil may be a good alternative in the liquid fuel production chain [18,20]. Sugarcane molasses have been successfully assessed by different researchers as supplements to be added to other compounds or to be used by themselves as the main components in the production of SCO [18,20,21], especially in processes involving the fungal species *Mucor circinelloides* URM 4182 and the freshwater *Scenedesmus obliquus*, and they were also chosen for this study due to their robustness, with characteristics that are conducive to easy growth and maintenance, as well as being good producers of SCO [22,23,24]. The use of SM as carbon sources in microbial processes may contribute to the feasibility and establishment of complex biorefineries, since they are a low-cost by-product from the well-established global sugarcane industry [18].

In this sense, the present study brings together two oleaginous strains, *M. circinelloides* and *S. obliquus*, in which the microalgae culture medium was modified with the addition of sugarcane molasses. Moreover, this paper addresses a sustainable alternative for the harvesting of microalgae cells via the employment of a mature fungal mycelium as a biosupport for cells’ harvesting, which eliminates the use of chemical products (associated with harvesting by sedimentation) and helps to reduce the high energy consumption, which is intrinsic to operations such as filtration and centrifugation, through the use of a fungal mycelium as a biological support for cell harvesting.

## 2. Materials and Methods

### 2.1. Microorganisms and Culture Medium

The filamentous fungus strain *Mucor circinelloides* f. *griseo-cyanus* URM 4182 was obtained at Federal University of Pernambuco, Recife, Brazil, from the URM Bank, and was maintained using a medium made with Potato Dextrose Agar (PDA), as described by [25]. The strain of *Scenedesmus obliquus* CCMA-UFSCar 604—freshwater microalgae—was provided by the Federal University of São Carlos, Brazil, and maintained in small static cultures in WC medium, under 20 ± 1 °C, renewed monthly.

For the fungal strains, incubation culture medium A [26] was used, composed of: glucose (2 g·L^−1^), KNO_3_ (1 g·L^−1^), KH_2_PO_4_ (0.075 g·L^−1^), K_2_HPO_4_ (0.1 g·L^−1^), MgSO_4_.2H_2_O (0.5 g·L^−1^), Ca(NO_3_)_2_.4H_2_O (0.0625 g·L^−1^), FeSO_4_.7H_2_O (0.01 g·L^−1^), yeast extract (0.5 g·L^−1^). The metal solution (1 mL.L^−1^) was composed of: H_3_BO_3_ (2.86 g·L^−1^), Na_2_MoO_4_.2H_2_O (0.39 g·L^−1^), ZnSO_4_.7H_2_O (0.22 g·L^−1^), MnCl_2_.4H_2_O (1.81 g·L^−1^), CuSO_4_.5H_2_O (0.079 g·L^−1^) and Co(NO_3_)_2_.6H_2_O (0.049 g·L^−1^). 

WC culture medium [27] was used for microalgae cells, composed of: CaCl_2_.2H_2_O (36.76 mg·L^−1^), MgSO_4_.7H_2_O (36.97 mg·L^−1^), NaHCO_3_ (12.60 mg·L^−1^), K_2_HPO_4_ (8.71 mg·L^−1^), NaNO_3_ (85.01 mg·L^−1^). The metal solution was composed of: Na_2_EDTA (4.36 mg·L^−1^), FeCl_3_.6H_2_O (3.15 mg·L^−1^), CuSO_4_.5H_2_O (0.01 mg·L^−1^), ZnSO_4_.7H_2_O (0.022 mg·L^−1^), CoCl_2_.6H_2_O (0.01 mg·L^−1^), MnCl_2_.4H_2_O (0.18 mg·L^−1^), Na_2_MoO_4_.2H_2_O (0.006 mg·L^−1^), H_3_BO_3_ (1.0 mg·L^−1^). The vitamin solution consisted of Thiamine (0.1 mg·L^−1^), Biotin (0.5 µg·L^−1^) and Cobalamin (0.5 µg·L^−1^).

### 2.2. Sugarcane Molasses

Sugarcane molasses were evaluated as a means of supplementation for microalgae growth and were provided by Mellaço de Cana (Saltinho, SP, Brazil). Their main constituents were sucrose (39.9%), fructose (7.5%), glucose (5.5%) and minor minerals, as detailed by Bento et al., 2020 [18]. The sugarcane molasses were added directly to the microalgae culture medium, at a concentration of 0.6 g for each 1 L of culture medium.

### 2.3. Lichenization Procedures—Microalgae and Fungus Consortium

For the isolated cultures of the microalgae and the filamentous fungus, two different orbital shakers were used, with an individual volume of 50 mL of culture medium in each flask and an incubation time of 72 h for both microorganisms. Fungal cultures were performed at 250 rpm, 26 ± 1 °C, and microalgae cultures at 140 rpm, 24 ± 1 °C. For microalgae growth, the orbital shaker was adapted with white LED illumination (150 μmol·m^−2^·s^−1^). For each of the isolated cultures, a total of 0.9 L of culture medium was prepared.

Each flask of culture medium A was inoculated with 4.25 × 10^5^ spores and, for microalgae cultivation, each flask of culture medium WC received 1.275 × 10^8^ microalgae cells. After this time, each of the fungal-grown mycelia (0.05 g dry weight) were harvested by filtration and added to a single 250-mL flask containing microalgae cell culture and incubation was continued for 108 h; at the end of this period, the biomass of the consortia was harvested by filtration.

**Figure 1:**
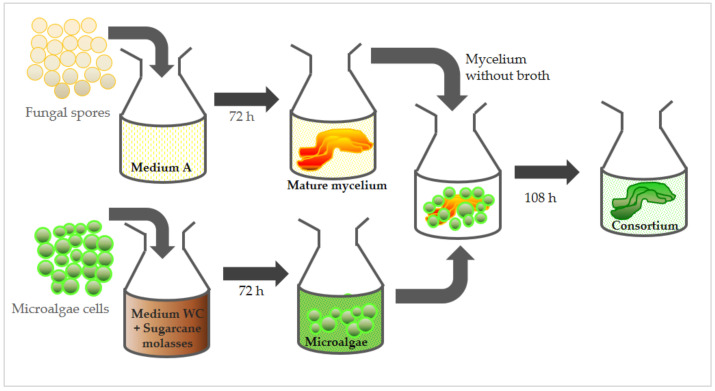
Scheme with sequence of steps for the formation of an algae-fungus consortium with *S. obliquus* and *M. circinelloides* strains.

In another sequence of new assays, 0.54 g sugarcane molasses was added to the total WC culture medium (0.9 L) and completely dissolved, with no change in the final volume. The culture medium was then divided among all flasks prior to the 1.275 × 10^8^ microalgae cells’ inoculation. The protocols for the spores’ cultivation were maintained in the same manner as described previously. Again, flasks were incubated separately for 72 h, in different orbital shakers, at the same conditions employed in the first group of assays. After 72 h of growth in isolation, each filtrated fungal-grown mycelium (approx. 100 mg) was added to a single flask containing microalgae culture (50 mL), and these remained incubated together until the end of the 108 h period. After this period, consortium biomass was harvested by filtration. All analyses and cultures were carried out in triplicate. Figure 1 briefly illustrates the sequence described for the formation of the microbial consortium, highlighting the supplementation step conducted using sugarcane molasses.

### 2.4. Analytical Methods

Both the fungus and consortium were harvested from their broth by filtration. An infrared-coupled balance (MOC63u, Shimadzu) was used to determine the cells’ dry weight. The phenol-sulfuric acid colorimetric method was applied to estimate the total sugar concentration [28].

Microalgal biomass in the consortium samples was determined according to the chlorophyll-A (Chl-a) content in the samples [26]. For that purpose, biomass samples were submitted to extraction by 5 mL of 90% *v*/*v* methanol solution under stirring at 150 rpm at 25 ± 1 °C, followed by the addition of 5 mL of distilled water. The chlorophyll extract present in the liquid phase was then filtered using a 0.45 μm syringe filter. To perform the determination of the Chl-a concentration, a UV-VIS spectrophotometer (Varian Cary 5000, Agilent Technologies, Santa Clara, CA, USA) was used. Calculations were made by a calibration curve considering the correlations of the algae’s dry weight and 665 nm Chl-a absorbance. After that, the fungal biomass present in the consortium was estimated according to the gravimetric difference between the consortium and the microalgae biomass weight.

Details of the physical interaction between the microalgae and the filamentous fungus were studied with a Hitachi TM 3000 (Hitachi High-Technologies Corporation, Tokyo, Japan) tabletop scanning electron microscope (SEM). For this analysis, the consortium biomass was dried under vacuum desiccator to constant weight. Then, residual moisture was removed by keeping the sample in an oven at 100 °C for 60 min. The dry biomass samples were metalized with silver and taken to the scanning electron microscope. 

Total lipids were quantified by 96% *v*/*v* ethanol extraction performed in a microwave irradiation reactor (CEM Model Discover DU-8081, CEM Corporation, NC, USA), at a temperature of 60 °C, during three cycles of 30 min (Carvalho et al., 2015). The composition of fatty acids was determined by an adapted version of the AOCS Ce 1–62 method via methylation using a methanol/BF_3_ mixture. Fatty acid methyl esters (FAMEs) were then identified by a flame-ionization detector gas chromatograph (GC Clarus 580, Perkin Elmer Inc., Waltham, MA, USA) using a 30 m capillary column (5% diphenyl, 95% dimethylpolysiloxane stationary phase) with a 0.25 mm internal diameter. Nitrogen was the carrier gas (1 mL·min^−1^). Methyl esters were identified through the use of a mixture standard for esters (MIX Supelco, Sigma-Aldrich, Bellefonte, PA, USA, FAME C6:0-capric acid to C24:0-lignoceric acid) and quantified in relative terms via the normalization of calculated peak areas.

### 2.5. Calculation of Biochemical Parameters Based on Cultivation Data

Both biomass and lipids were evaluated in terms of their concentration and productivity. The production efficiency of microbial lipids was obtained based on biomass (*X*) and lipid (*P*) concentrations, as well as lipid yield (%*P*), as shown in Equations (1)–(3), respectively. The results were also analyzed by considering the volumetric productivity (expressed in mg·L^−1^·day^−1^) of biomass (*Qx*) and lipid (*Qp*), as shown in Equations (4) and (5), which considers the biomass (*X*) and the lipid (*P*) concentrations at the end of cultivation (*f*) as well as at the beginning:

(1) $$X=\frac{dry\;weight\;biomass\;\left(mg\right)}{volume\;of\;culture\;\left(L\right)}$$

(2) $$P=\frac{total\;lipids\;\left(mg\right)}{volume\;of\;culture\;\left(L\right)}$$

(3) $$\%P=\frac{lipids\;mass\;\left(mg\right)}{dry\;weight\;biomass\;\left(mg\right)}\times 100$$

(4) $$Qx=\frac{\Delta X}{\Delta t}=\frac{X_{f}-X_{i}}{t_{f}-t_{i}}$$

(5) $$Qp=\frac{\Delta P}{\Delta t}=\frac{P_{f}-P_{i}}{t_{f}-t_{i}}$$

in Equations (4) and (5): *_f_* means final and *i* means initial.

### 2.6. Recovery Efficiency of Microalgae Cells

Once the microalgae could grow free in the medium, unattached to the consortium, the recovery efficiency (RE) of the microalgae cells was defined according to equation 6, where *m* indicates the dry biomass weight (mg) of the consortium, *w* is the mass of the microalgae fraction over the total biomass weight, [microalgae] means the concentration (mg·L^−1^) of algae suspended in the medium, and *V* is the medium volume expressed in L.

(6) $$RE\;\left(\%\right)=\frac{m_{consortium}\cdot w_{microalgae}}{m_{consortium}\cdot w_{microalgae}+\left[microalgae\right]\cdot V}\cdot 100\%$$

## 3. Results and Discussion

### 3.1. Growth Parameters of Microbial Species and Consortia, and Physical Characterization

Both microorganisms evaluated herein have been widely described as lipid-producer microorganisms [29,30]. This requirement is interesting, as it makes these species promising for industrial applications, such as the generation of biodiesel raw materials or fine chemical intermediates. Table 1 summarizes the biochemical cultivation parameters obtained for the microalgae and the respective results for the consortia formed without/with the supplementation of sugarcane molasses. The mycelium of the filamentous fungus was employed as a biosupport; therefore, sugarcane molasses were not supplemented in fungi culture medium, and consequently, there were no variations related to the fungi’s biochemical parameters and the sugarcane molasses.

The results herein demonstrated that the lipids increased by 3.5 times without the use of molasses in the lichen biomasses and by 7.5 times when the molasses were used, as compared to the fungal strain parameters. In terms of the lipid-specific yield (%), this increase was 3.2 and 5.3 times, respectively, in relation to the fungal strain parameters, achieving 23% and 38% dry biomass weight, respectively.

The volumetric biomass production rate (*Qx*) results demonstrate that growth rates for the evaluated microorganisms were different. Since the fungal strain grows faster, higher values of *Qx* for the fungus than for the microalgae in the culture could be expected, and this in fact occurred in cultures where the microalgae was cultivated without the supplementation of sugarcane molasses in the culture medium. On the other hand, the growth rates for the microalgae were higher than those for the fungus, when the algae cultures were carried out with supplementation of sugarcane molasses, since, under these conditions, the cultivation of algae becomes mixotrophic, with the consumption of both CO_2_ and organic carbon sources. The low glucose concentration in the fungal growth medium (2 g·L^−1^) did not provide the ideal C/N ratio for fungal lipid production. The carbon source was almost totally consumed within 24 h of incubation, which explains the result of 7.2 ± 0.4% in relation to the dry biomass weight, which was much lower than other values reported in the literature [20,21]. On the other hand, it was noted that the WC culture medium proved to be adequate for the accumulation of lipids by the *S. obliquus* species, the lipid yield of which reached 30 ± 0.4% in isolated culture. Furthermore, the supplementation of sugarcane molasses in the cultivation of algae notably favored both biomass (1042 mg·L^−1^ algae/1270 mg·L^−1^ lichen) and lipid accumulation (322 mg·L^−1^ algae/482.6 mg·L^−1^ lichen) by the microalgae strains and by the consortium, in comparison to the cultivations without SM. The positive influence of sugarcane molasses as a means of supplementation to enhance biomass and lipid production for a strain of this fungal species was demonstrated by Bento el al., 2020 [18]. 

In the present study, the focus was on the formation of a consortium between algae and fungi, where the microalgae were the vector for accumulation of biomass and lipids and the filamentous fungus mycelium served as a biosupport for the anchorage of microalgae cells. Microalgae are able to synthesize and accumulate lipids in their cells for energy storage. Those lipids are biosynthesized by the accumulation of NADH and L-α-phosphoglycerol derived from the glycolytic pathway, which happens when citrate enzyme activity is inhibited by a low photosynthetic rate or low nitrogen availability, preventing acetyl CoA from reacting in the TCA cycle. The accumulated acetyl CoA activates the respective carboxylase enzyme, causing the transfer of acyl residues that act together with L-acylglycerol-3-phosphate acyltransferase, generating malonyl CoA and L-α-phosphatic acid, as well as further phospholipids, diacylglycerols and triacylglycerols [31,32]. 

With the supplementation of the culture medium with sugarcane molasses, cell growth became mixotrophic, and, in addition to light for photosynthesis, the algal species metabolized carbon from sugars that were present in sugarcane molasses. Total sugars in the culture broth just before incubation, with the addition of sugarcane molasses to the WC culture medium, amounted to 0.360 g·L^−1^. After 180 h of incubation, the remaining sugars totaled 0.013 g·L^−1^, which confirmed that practically all sugar had been consumed. The fact that the cultivation of microalgae using sugarcane molasses showed an increase in the biomass growth (Table 1) indicates that the majority of added sugars were assimilated by the microalgae species, contributing to the biomass and lipid production in the consortium.

**Figure 2:**
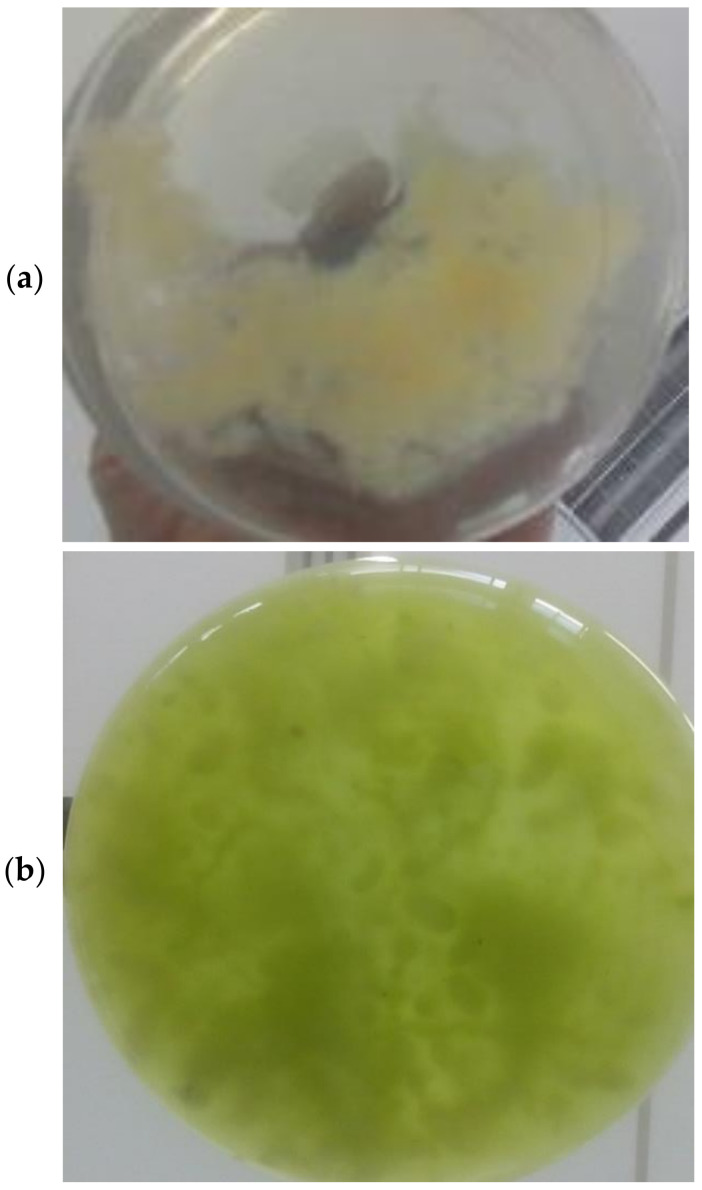

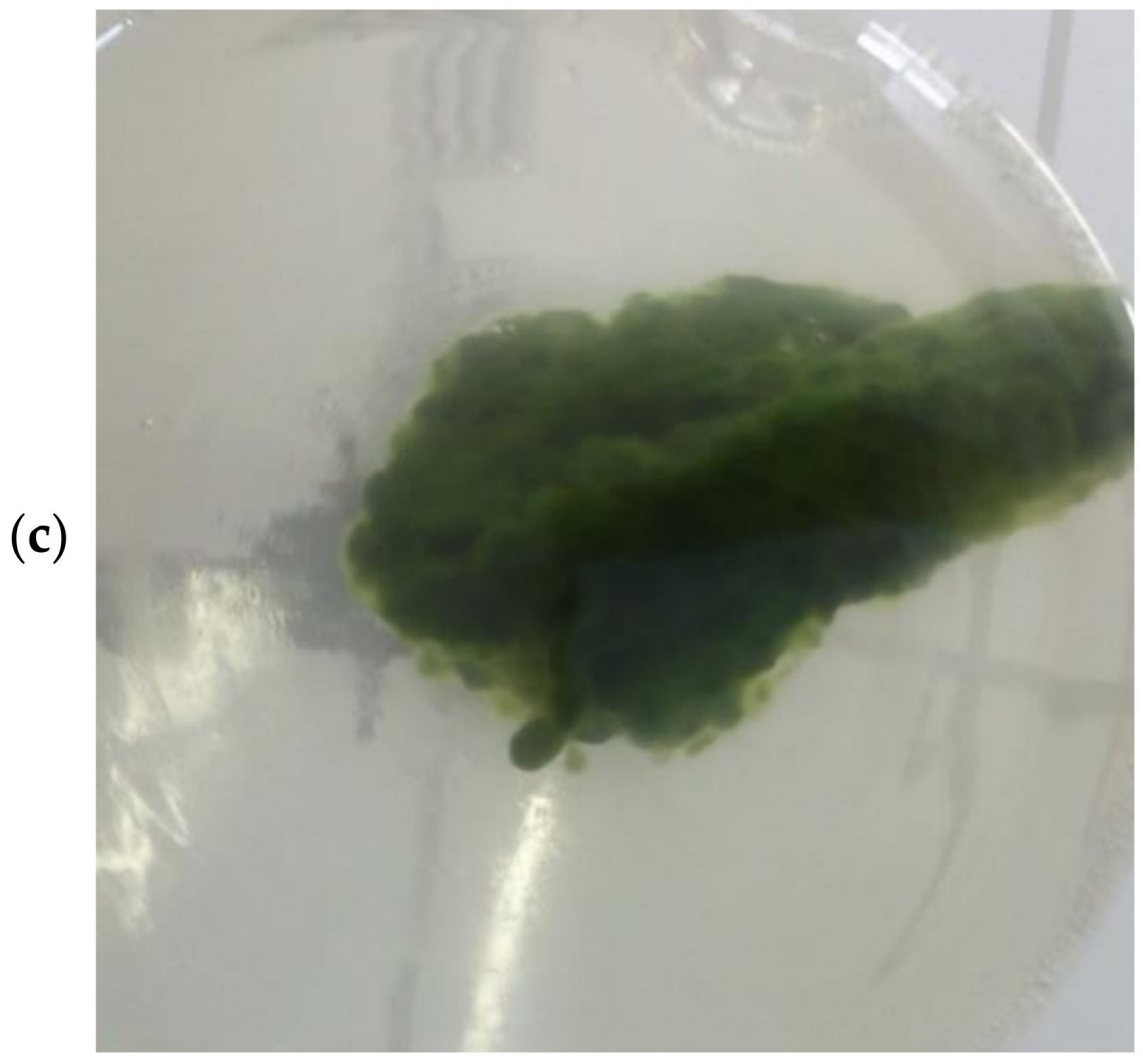
Lichen formation steps: (**a**) solo fungal mycelium; (**b**) fungal mycelium added to algae culture; (**c**) lichen-like structure after 108 h with algae cells adhering to fungal mycelium.

Figure 2 shows images of the lichening formation steps; Figure 2a displays the solo fungal mycelium, Figure 2b displays the fungal mycelium added to the algae medium, and Figure 2c displays the formation of the lichen-like structure after 108 h, where it is possible to visually notice that most of the algal cells adhered to the fungal hyphae/mycelium and a high harvesting efficiency was achieved.

### 3.2. Lipid Characteristics: Fatty Acid Profile

The results presented in Table 2 describe the fatty acid composition of microbial lipids from the microalgae *S. obliquus*, with and without supplementation of sugarcane molasses, and also the lipid fatty acid profile of the consortium generated between the microalgae and the filamentous fungus.

Regarding the composition of fatty acids obtained from biomass without SM supplementation, the microbial consortium presented a fatty acid profile ranging from C16 up to C18, with a balanced distribution among the saturated (SFA) and unsaturated (mono- and poly- MUFA and PUFA, respectively) types of fatty acids. The values obtained for the samples were SFA + MUFA = 49.7% and PUFA = 50.3%. The properties of saponifiable lipids and the products generated, such as biodiesel, are directly related to the fatty acid profile. For example, higher viscosity and density in biodiesel products are usually linked to higher degrees of saturation; on the other hand, unsaturated fatty acids, in abundance, can reduce the resistance of the biodiesel to oxidation [33]. Therefore, compared to that which was reported by other authors [33], for this distribution of fatty acids, a good balance between flow and viscosity properties was expected, as a result of the presence of unsaturated acids, as well as stable oxidation, due to the saturated fatty acids in the composition. 

Regarding the fatty acid composition for the consortium cultivated with SM supplementation, there was a smaller contribution of saturated fatty acids, but there was a greater contribution of monounsaturated fatty acids, due to the occurrence of palmitoleic acid in addition to oleic acid. In sum, SFA + MUFA amounted to a composition of 52.7%, while the polyunsaturated fatty acids totaled a composition of 47.4%. These values were almost close to the composition of fatty acids without supplementation of sugarcane molasses; however, in the group of polyunsaturated fatty acids from biomass supplemented with SM, a predominance of linolenic acid was observed, which occurred in a proportion that was greater than that of linoleic acid. Even in the group of polyunsaturated fatty acids, values that were very close to the fatty acid composition of the filamentous fungus lipids were noted. Thus, it could be noted that the lipid profile of the microalgae cultivated with SM also had a composition that was very close to the corresponding microbial consortium. Although the use of the supplementation of sugarcane molasses resulted in a substantial increase in biomass and total lipids, the fatty acid composition was not significantly different. These initial results suggest positively that the use of sugarcane molasses in co-cultivation is positive and deserves to be further explored.

### 3.3. Evaluation of Harvesting Efficiency and Consortium Biomass Composition 

Considering the use of the microalgae of the genus *Scenedesmus*, only a few reports have described its harvesting with fungal assistance (as summarized in Table 3). The lichen formation by *Scenedesmus* sp. and *Trichoderma reesei* QM 9414 was studied in [34], and showed a high harvesting efficiency of >94%. In another study [35], a harvesting of *Scenedesmus obliquus* SIT06 of up to 92.7% was achieved by *Cunninghamella echinulata* TPU 4652. A consortium obtained by *Scenedesmus quadricauda* and *Aspergillus fumigatus* also indicated a high harvesting efficiency (>90%) in a study evaluated by Wrede et al., 2014 [36].

The results obtained in this present study indicated a high harvesting potential of *S. obliquus* by *M*. *circinelloides*, achieving an efficiency of up to 99.7%. A similar result was found by Pei et al., 2021 [37] in a consortium composed of *Scenedesmus* sp. and *Aspergillus niger*, with a harvesting efficiency of 99.4%. These studies, in combination, are a great reference to suggest the potential of the bioprocess proposed herein to fulfill certain algae-fungi-based biorefinery needs, such as that of high harvesting efficiency combined with high lipid production. 

**Figure 3:**
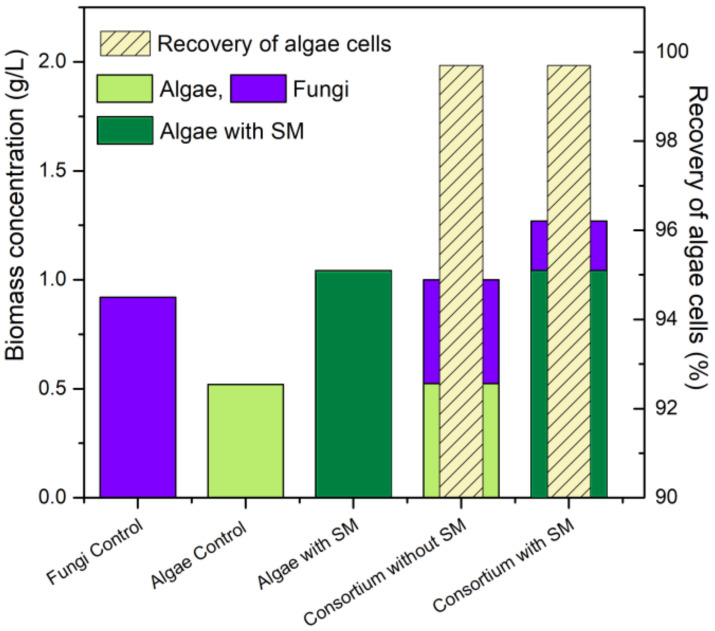
Summary of results according to biomass accumulation and distribution fungi (blue), algae control (light green), algae with SM (dark green) and % recovery of algae cells (striped).

The results obtained in this work are detailed in Figure 3, where it can be seen that the addition of sugarcane molasses favored the increase in algae biomass in the microalgae and consortium cultivations, achieving equal harvesting efficiency in the consortia with and without sugarcane molasses (99.7%). These data suggest a potential integration of sugarcane agroindustry in sustainable algae-based biorefineries, through which it may be possible to promote novel outstanding alternatives for integrated efficient bioprocesses.

### 3.4. Formation of the Lichen-like Structure at the Molecular Level 

The filamentous fungus biomass is composed of a tangle of intertwined hyphae, which constitute the mycelium. At a molecular level, once algae cells and fungal mycelium can grow together, different interactions may take place between the microalgae cells and the fungal hyphae, so that the lichen-like structure is not only formed by the entrapment of microalgae cells in the mycelium network.

First, both microorganisms can benefit each other through gas and nutrient exchange. During the joint incubation period, the carbon dioxide (CO_2_) released by the fungi is possibly absorbed by the microalgae cells in the photosynthesis process, which may remove the need of further CO_2_ aeration. On the other hand, the oxygen (O_2_) released by the microalgae cells can be absorbed by the filamentous fungi and further used for energy generation and biomass growth [26,38].

In addition, the organic acids released by the fungus, which may inhibit their growth in the later stages of its own metabolism, are absorbed by the microalgae, contributing to their growth [38]. For example, in a co-culture study carried out by Zhang et al. [39] with the species *Chlorella vulgaris* and *Rhodotorula glutinis*, a decrease in the levels of propionic acid, pyruvic acid, acetic acid and palmitic acid was highlighted, while the levels of glycine and proline increased, indicating that the microalgae consumed the organic acids released by the yeast. The authors also demonstrated that there was an increase in pH in the alga-yeast culture medium, compared to the monocultures. According to Xue et al. [40] fungal species are able to cleave several complex sugars into simple ones, which can then be used by microalgae for their cell multiplication.

While microalgae convert CO_2_ into bicarbonate for their own consumption, the culture medium becomes alkaline through the release of OH^−^ ions [40]. This alkalinity is beneficial for many fungi, as it balances the acidity of the culture medium, which interferes with the growth of the fungal species. Thus, the interaction of metabolites can result in the balance of intrinsic O_2_/CO_2_, pH and dissolved oxygen in the medium, leading to an increase in the growth rate and metabolite production for both species [38,39,40], which can be noticed by the increase in biomass and the accumulation of lipids, in line with the results found in this work.

Furthermore, the biomass surface of the lichen that is formed is soft and covered with flexible, hydrated macromolecules, which facilitates adhesion between cells and from cells to surfaces [10]; in other words, the macromolecules contained in the cells of microalgae and fungi are interconnected.

According to Reis et al. [41], specifically on a lichen-like structure of fungi and algae, the formation of such a complex structure may consist of any of the following mechanisms: first, the adhesion of fungal spores or the germination of fungal spores and algal cells in a matrix; second, the proliferation of mononuclear cells on the surface or the matrix; and third, the induction of the formation of fungal hyphae, promoting the fixation of algae in the mycelia [41]. The present work is aligned with the third mechanism, since the mycelium of the fungus (containing the branches of hyphae) was used as a biosupport, that is, it became the matrix itself for the aggregation or fixation of the microalgae cells.

**Figure 4:**
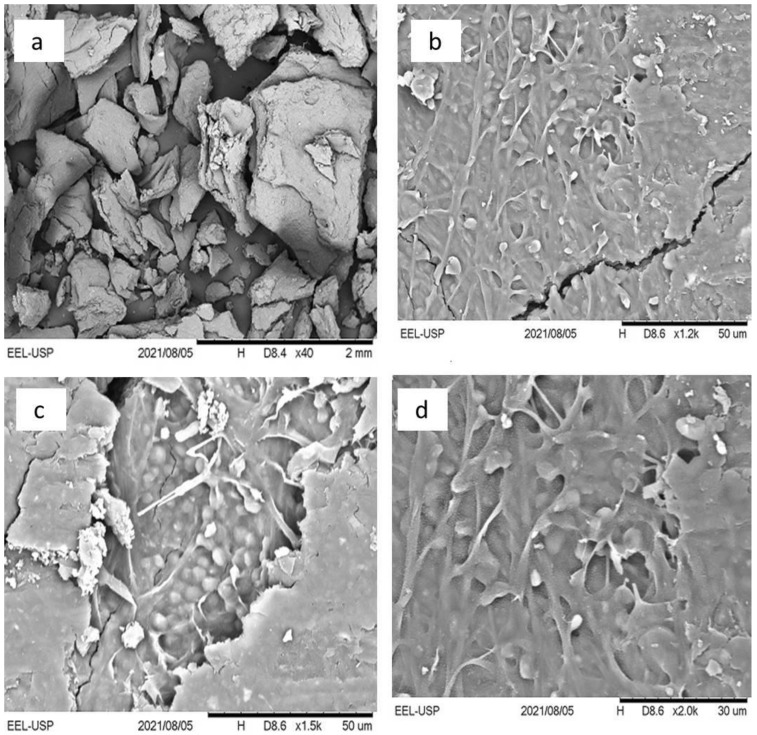
Scanning electron microscope pictures of the lichen-like structure composed of *M. circinelloides* mycelium and *S. obliquus* cells at different magnifications: (**a**) 40×, (**b**) 1200×, (**c**) 1500×, (**d**) 2000×. In (**a**), the image of a biomass grain of the lichen-like structure can be observed. In (**b**–**d**), the fixation of algae cells (small drop shapes with white color) and fungal hyphae (in branch format with light gray color) can be well visualized. In (**c**) specifically, there are clumps of algae cells in certain places where the branch fungal mycelium also predominates and forms a nest structure. In (**c**,**d**), it can be observed that some algae are interconnected among filamentous fungi.

According to Gultom and Hu [10], fungal spores have crystal-like layers, which prevent aggregation within themselves and with other strains; however, growing hyphae act as the main promoter of interconnections between germinating spores and other species. Microscopically, lectin carbohydrate is an important factor in the binding of algae and fungi [10,41,42]. The presence of calcium ions, which act as a link between the negatively charged cells and extracellular nucleic acids and, thus, allow cell-to-cell communication through electrostatic interactions, is particularly involved in the role of the lectin-carbohydrate binding [42]. Through mechanisms that are still unclear, weak lectin-carbohydrate binding forms stable cellular arrangements and causes increased shear strength [41,42]; therefore, cells and mycelium remain linked, forming a unique microbial consortium biomass. The microphotograph in Figure 4 shows the structure of the lichen-like structure composed of *S. obliquus* and *M. circinelloides* that was seen by scanning electron microscope (SEM); this structure was found to contain several microalgae cells that adhered to the fungal hyphae.

### 3.5. Potential Membrane-Related Applications and Perspectives

A lichen-like strategy for microalgae cultivation shows an optimistic scaling-up potential once its bioreactor cultivation can be related to membrane-bioreactors, whereas the lichen acts as a natural membrane for solid–liquid separation, which opens up the possibility of wastewater treatment and valorization alongside simultaneous low-cost algae harvesting. Studies have indicated the potential of combining algae cultivation and membrane-based technologies (membrane bioreactors, forward osmosis, ceramic membranes and others), which has resulted in efficient harvesting and bioremediation systems [43,44,45,46,47].

Filamentous fungi-microalgae consortia have the advantage of presenting both species bioremediation properties as well as physical structures that may act as biomembranes. The potential of lichens’ nutrient removal, aligned simultaneously with low-cost filtration, may pave the way for the development of novel environmentally friendly technologies that can contribute to the feasible establishment of fungal-microalgal biorefineries and the valorization of wastewater and its by-products.

## 4. Conclusions

The microorganisms *S. obliquus* and *M. circinelloides* composed a lichen-type structure that resulted in a unique lipid-rich biomass (38.2% in weight), in which a mature fungal mycelium played the role of a biosupport to microalgae cells. The supplementation of the algae growth medium with SM contributed to the increasing of the biomass and lipid content in this structure. A balanced fatty acid composition, potentially suitable as a feedstock for the production of biodiesel and other value-added bioproducts, as well as an eco-friendly alternative to algal biomass harvesting (99.7% efficiency) was presented. The physical interactions between microorganisms forming a lichen-like structure were confirmed macro- and microscopically. This lichen-like structure also showed the potential to act as a natural membrane.

## Figures and Tables

**Table 1:** Biochemical parameters resulting of the cultivation of the strains microalgae *S. obliquus,* fungus *M. circinelloides* and their consortium—without and with the addition of sugarcane molasses (SM) to the microalgae culture medium.

Parameters	Cultivation without SM		Cultivation with SM		Filamentous Fungus
	Consortium	Microalgae	Consortium	Microalgae	
*X* (mg·L^−1^)	1000 ± 0.7	524 ± 0.7	1270 ± 0.7	1042 ± 0.7	900 ± 0.5
*P* (mg·L^−1^)	230.0 ± 0.3	149.2 ± 0.3	482.6 ± 0.3	322.0 ± 0.3	64.8 ± 0.3
*P* (%)	23.0 ± 0.4	28.5 ± 0.3	38.2 ± 0.5	30.9 ± 0.4	7.2 ± 0.3
*Q_X_* (mg·L^−1^·day^−1^)	133.3 ± 0.4	69.8 ± 0.4	169.3 ± 0.4	138.9 ± 0.4	120 ± 0.4
*Q_P_* (mg·L^−1^·day^−1^)	30.7 ± 0.4	19.9 ± 0.4	64.4 ± 0.5	42.9 ± 0.4	8.6 ± 0.4

**Table 2:** Fatty acid composition of lipid material from the biomasses of microalgae (*S. obliquus*), fungus (*M. circinelloides)* and lichens grown in an orbital shaker for 7.5 days.

Fatty Acid Profile		Cultivation without SM		Cultivation with SM		Filamentous Fungus
		Consortium	Microalgae	Consortium	Microalgae	
C8:0	Caprylic acid	3.7 ± 0.17	10.7 ± 0.18	0.4 ± 0.25	0.12 ± 0.25	18.1 ± 0.23
C16:0	Palmitic acid	30.7 ± 0.15	31.7 ± 0.30	18.9 ± 0.18	33.1 ± 0.18	24.0 ± 0.29
C18:0	Stearic acid	0	0	1.6 ± 0.18	1.49 ± 0.18	4.2 ± 0.17
SFA	Saturated Fatty Acids	34.4	42.4	20.9	34.7	46.3
C16:1	Palmitoleic acid	0	0	9.7 ± 0.18	1.23 ± 0.25	0
C18:1	Oleic acid	15.3 ± 0.22	13.5 ± 0.31	22.1 ± 0.31	30.56 ± 0.30	0
MUFA	Monounsaturated Fatty Acids	15.3	13.5	31.8	31.7	0
C18:2	Linoleic acid	31.1 ± 0.20	36.9 ± 0.18	16.1 ± 0.25	12.5 ± 0.30	22.9 ± 0.16
C18:3	Linolenic acid	19.2 ± 0.20	7.2 ± 0.18	31.3 ± 0.25	21.0 ± 0.30	30.8 ± 0.27
PUFA	Polyunsaturated Fatty Acids	50.3	44.1	47.4	33.5	53.7

**Table 3:** Relative harvesting efficiencies of microalgae-fungi consortium described in the literature and the present study.

Microalgae Species	Fungal Species	Harvesting Efficiency	Reference
*Scenedesmus* sp.	*Trichoderma reesei* QM 9414	>94%	[34]
*S. obliquus* SIT06	*Cunninghamella echinulata* TPU 4652	92.7%	[35]
*S. quadricauda*	*Aspergillus fumigatus*	>90%	[36]
*Scenedesmus*sp.	*Aspergillus niger*	99.4%	[37]
*S. obliquus* CCMA-UFSCar 604	*M. circinelloides* URM 4182	99.7%	This study

## Data Availability

Supporting data can be acquired privately from the authors or any member of the Biocatalysis Research Group of the Engineering School of Lorena (EEL-USP).

## References

[1] Zhu L. (2015). Biorefinery as a promising approach to promote microalgae industry: An innovative framework. Renew. Sustain. Energy Rev..

[2] Liyanaarachchi V.C., Premaratne M., Ariyadasa T.U., Nimarshana P.H.V., Malik A. (2021). Two-stage cultivation of microalgae for production of high-value compounds and biofuels: A review. Algal Res..

[3] Pan M., Zhu X., Pan G., Angelidak I. (2021). Integrated valorization system for simultaneous high strength organic wastewater treatment and astaxanthin production from *Haematococcus pluvialis*. Bioresour. Technol..

[4] Agarwal A., Mhatre A., Pandit R., Lali A.M. (2021). Synergistic biorefinery of *Scenedesmus obliquus* and *Ulva lactuca* in poultry manure towards sustainable bioproduct generation. Bioresour. Technol..

[5] Zuccaro G., Del Mondo A., Pinto G., Pollio A., De Natale A. (2021). Biorefinery-based approach to exploit mixed cultures of *Lipomyces starkeyi* and *Chloroidium saccharophilum* for single cell oil production. Energies.

[6] Chisti Y. (2007). Biodiesel from microalgae. Biotechnol. Adv..

[7] Visigalli S., Barberis M.G., Turolla A., Canziani R., Zrimec M.B., Reinhardt R., Ficara E. (2021). Electrocoagulation–flotation (ECF) for microalgae harvesting—A review. Sep. Purif. Technol..

[8] Das P.K., Rani J., Rawat S., Kumar S. (2021). Microalgal co-cultivation for biofuel production and bioremediation: Current status and benefits. BioEnergy Res..

[9] Pragya N., Pandey K.K., Sahoo P.K. (2013). A review on harvesting, oil extraction and biofuels production technologies from microalgae. Renew. Sustain. Energy Rev..

[10] Gultom S., Hu B. (2013). Review of microalgae harvesting via co-pelletization with filamentous fungus. Energies.

[11] Reis C.E.R., Bento H.B.S., Carvalho A.K.F., Rajendran A., Hu B., De Castro H.F. (2019). Critical applications of *Mucor circinelloides* within a biorefinery context. Crit. Rev. Biotechnol..

[12] Zhang J., Hu B. (2012). A novel method to harvest microalgae via co-culture of filamentous fungi to form cell pellets. Bioresour. Technol..

[13] Li T., Li C.-T., Butler K., Hays S.G., Guarnieri M.T., Oyler G.A., Betenbaugh M.J. (2017). Mimicking lichens: Incorporation of yeast strains together with sucrose-secreting cyanobacteria improves survival, growth, ROS removal, and lipid production in a stable mutualistic co-culture production platform. Biotechnol. Biofuels.

[14] Olson D.G., Mc Bride J.E., Shaw A.J., Lynd L.R. (2012). Recent progress in consolidated bioprocessing. Curr. Opin. Biotechnol..

[15] Loures C., Amaral M.S., Da Rós P.C.M., Zorn S.M.F.E., De Castro H.F., Silva M.B. (2018). Simultaneous esterification and transesterification of microbial oil from *Chlorella minutissima* by acid catalysis route: A comparison between homogeneous and heterogeneous catalysts. Fuel.

[16] Zorn S.M.F.E., Reis C.E.R., Bento H.S.B., Carvalho A.K.F., Silva M.B., De Castro H.F. (2020). In situ transesterification of marine microalgae biomass via heterogeneous acid catalysis. BioEnergy Res..

[17] Jiru T.M., Steyn L., Pohl C., Abate D. (2018). Production of single cell oil from cane molasses by *Rhodotorula kratochvilovae* (syn, *Rhodosporidium kratochvilovae*) SY89 as a biodiesel feedstock. Chem. Cent. J..

[18] Bento H.B.S., Carvalho A.K.F., Reis C.E.R., De Castro H.F. (2020). Single cell oil production and modification for fuel and food applications: Assessing the potential of sugarcane molasses as culture medium for filamentous fungus. Ind. Crop. Prod..

[19] Tinôco D., Castro A.M., Seldin L., Freire D.M.G. (2021). Production of (2R,3R)-butanediol by *Paenibacillus polymyxa* PM 3605 from crude glycerol supplemented with sugarcane molasses. Process. Biochem..

[20] Reis C.E.R., Valle G.F., Bento H.B.S., Carvalho A.K.F., Alves T.A., De Castro H.F. (2020). Sugarcane by-products within the biodiesel production chain: Vinasse and molasses as feedstock for oleaginous fungi and conversion to ethyl esters. Fuel.

[21] Carvalho A.K.F., Bento H.B.S., Reis C.E.R., De Castro H.F. (2019). Sustainable enzymatic approaches in a fungal lipid biorefinery based in sugarcane bagasse hydrolysate as carbon source. Bioresour. Technol..

[22] Darki B.Z., Seyfabadi J., Fayazi S. (2017). Effect of nutrients on total lipid content and fatty acids profile of *Scenedesmus obliquus*. Braz. Arch. Biol. Technol..

[23] Cheung S.L., Allen D.G., Short S.M. (2020). Specific quantification of *Scenedesmus obliquus* and *Chlorella vulgaris* in mixed-species algal biofilms. Bioresour. Technol..

[24] Loganathan B.G., Orsat V., Lefsrud M., Wu B.S. (2020). A comprehensive study on the effect of light quality imparted by light-emitting diodes (LEDs) on the physiological and biochemical properties of the microalgal consortia of *Chlorella variabilis* and *Scenedesmus obliquus* cultivated in dairy wastewater. Bioprocess. Biosyst. Eng..

[25] Carvalho A.K.F., Rivaldi J.D., Barbosa J.C., De Castro H.F. (2015). Biosynthesis, characterization and enzymatic transesterification of single cell oil of *Mucor circinelloides*—A sustainable pathway for biofuel production. Bioresour. Technol..

[26] Rajendran A., Hu B. (2016). Mycoalgae biofilm: Development of a novel platform technology using algae and fungal cultures. Biotechnol. Biofuels.

[27] Guillard R.R., Lorenzen C.J. (1972). Yellow-green algae with chlorophyllide C1,2. J. Phycol..

[28] Dubois M., Gilles A.K., Hamilton K.J., Rebers A.P., Smith F. (1956). Colorimetric method for determination of sugars and related substances. Anal. Chem..

[29] Xia C., Zhang J., Zhang W., Hu B. (2011). A new cultivation method for microbial oil production: Cell pelletization and lipid accumulation by *Mucor circinelloides*. Biotechnol. Biofuels.

[30] Tran D.T., Yeh K.L., Chen C.L., Chang J.S. (2012). Enzymatic transesterification of microalgal oil from *Chlorella vulgaris* ESP-31 for biodiesel synthesis using immobilized *Burkholderia* lipase. Bioresour. Technol..

[31] Huang G., Chen F., Wei D., Zhang X., Chen G. (2010). Biodiesel production by microalgal biotechnology. Appl. Energy.

[32] Singh P., Kumari S., Guldhe A., Misra R., Rawat I., Bux F. (2016). Trends and novel strategies for enhancing lipid accumulation and quality in microalgae. Renew. Sustain. Energy Rev..

[33] Talebi A.F., Motashami S.K., Tabatabaei M., Tohidfar M., Bagheri A., Zeinalabedini M., Mirzaei H.H., Mirzajanzadeh M., Shafaroudi S.M., Bakhtiari S. (2013). Fatty acids profiling: A selective criterion for screening microalgae strains for biodiesel production. Algal Res..

[34] Srinuanpan S., Chawpraknoi A., Chantarit S., Cheirsilp B., Prasertsan P. (2018). A rapid method for harvesting and immobilization of oleaginous microalgae using pellet-forming filamentous fungi and the application in phytoremediation of secondary effluent. Int. J. Phytoremediation.

[35] Srinuanpan S., Cheirsilp B., Prasertsan P., Kato Y., Asano Y. (2018). Photoautotrophic cultivation of oleaginous microalgae and co-pelletization with filamentous fungi for cost-effective harvesting process and improved lipid yield. Aquac. Int..

[36] Wrede D., Taha M., Miranda A.F., Kadali K., Stevenson T., Ball A.S., Mouradov A. (2014). Co-cultivation of fungal and microalgal cells as an efficient system for harvesting microalgal cells, lipid production and wastewater treatment. PLoS ONE.

[37] Pei X.-Y., Ren H.-Y., Liu B.-F. (2021). Flocculation performance and mechanism of fungal pellets on harvesting of microalgal biomass. Bioresour. Technol..

[38] Arora N., Patel A., Mehtani J., Pruthi A.P., Pruthi V., Poluri M.K. (2019). Co-culturing of oleaginous microalgae and yeast: Paradigm shift towards enhanced lipid productivity. Environ. Sci. Pollut. Res..

[39] Zhang Z., Ji H., Gong G., Zhang X., Tan T. (2014). Synergistic effects of oleaginous yeast *Rhodotorula glutinis* and microalga *Chlorella vulgaris* for enhancement of biomass and lipid yields. Bioresour. Technol..

[40] Xue F., Miao J., Zhang X., Tan T. (2010). A new strategy for lipid production by mix cultivation of *Spirulina platensis* and *Rhodotorula glutinis*. Appl. Biochem. Biotechnol..

[41] Reis C.E.R., Rajendran A., Silva M.B., Hu B., De Castro H.F., Singh O., Chandel A. (2018). The application of microbial consortia in a biorefinery context: Understanding the importance of artificial lichens. Sustainable Biotechnology—Enzymatic Resources of Renewable Energy.

[42] Zamalloa C., Gultom S.O., Rajendran A., Hu B. (2017). Ionic effects on microalgae harvest via microalgae fungi co-pelletization. Biocatal. Agric. Biotechnol..

[43] Castro-Muñoz R., García-Depraect O. (2021). Membrane-based harvesting processes for microalgae and their valuable-related molecules: A review. Membranes.

[44] Ding Y., Wang S., Ma H., Ma B., Guo Z., You H., Mei J., Hou X., Liang Z., Li Z. (2021). Effect of different influent conditions on biomass production and nutrient removal by aeration microalgae membrane bioreactor (ICFB-MMBR) system for mariculture wastewater treatment. Membranes.

[45] Park K., Kim P., Kim H.G., Kim J. (2019). Membrane fouling mechanisms in combined microfiltration-coagulation of algal rich water applying ceramic membranes. Membranes.

[46] Haupt A., Lerch A. (2018). Forward osmosis application in manufacturing industries: A short review. Membranes.

[47] Drexler I.L., Yeh D.H. (2014). Membrane applications for microalgae cultivation and harvesting: A review. Rev. Environ. Sci. Bio/Technol..

